# Editorial: Bioethics Amidst the COVID-19 Pandemic

**DOI:** 10.3389/fmed.2021.778146

**Published:** 2021-11-26

**Authors:** Maysa Al-Hussaini, Asem H. Mansour, Thalia A. Arawi, Ma'n H. Zawati, Haavi Morreim

**Affiliations:** ^1^Human Research Participants Protection (HRPP) Office, King Hussein Cancer Center, Amman, Jordan; ^2^Faculty of Medicine, American University of Beirut, Beirut, Lebanon; ^3^Centre of Genomics and Policy, McGill University, Montreal, QC, Canada; ^4^University of Tennessee Health Science Center (UTHSC), Memphis, TN, United States

**Keywords:** bioethics, COVID-19, resource allocation, clinical trials, do not resuscitate (DNR), cancer, psychological well-being, informatics

March 2020 witnessed the WHO's declaration of COVID-19 as a global pandemic. It is a date that has imprinted history. Almost 2 years later and the pandemic remains in many ways a global mystery: how did it come to be? Will there be new waves and additional mutations? Is it man-made and thus a form of biological warfare or is it released to trick governments and people into buying vaccines to increase the profit of pharmaceutical companies with trillions of dollars that will be used to subsidize more wars? Almost 2 years now and a plethora of questions continue to be raised in connection to the vaccine: which one is better? Should there be a booster shot? Will it tamper our DNA? Does it really protect from the pandemic? Does it work with new variants?

Scarcity of resources, beds, and ventilators brought to light new moral conundrums for clinicians and ethicists: How should scarce medical resources be allocated? Should the elderly be sacrificed? Is a unilateral Do-Not-Resuscitate (DNR) acceptable from the moral standpoint? etc.

The above were enough for us to see the cruciality of delving more into bioethical concerns during pandemics which led to a call that we issued on May 2020 at the earliest stages of the pandemic. We received global contributions tackling different issues or similar ones from different cultural backgrounds. Their contributions made the content rich with information and triggered more thinking about ethical dilemmas and how to solve them. Most importantly, it made us realize how important bioethics is during pandemics globally and how ethical concerns became more central in medical care globally, regardless of the economic divide, cultural differences, and/or political ideologies.

The contributions were divided into six main entries/themes:

1. *Care offered to patients, regardless of their ailment*. As caring for patients suffering from cancer began as an enigma in the early stages of the pandemic, Al-Tabba et al. address this issue in their article calling for practical measures that are applicable in different settings and with different resource capacities basing their work on the experience of the King Hussein Cancer Center in Jordan.
  In their “Do-Not-Resuscitate and COVID-19; the Ethical Dilemma and Suggested Solutions,” Sultan et al. discuss the issue of whether physicians or the healthcare system can take a unilateral decision about withdrawal of life support in situations of resource scarcity. In their “The Care for non-COVID-19 Patients: A Matter of Choice or Moral Obligation?” Hassan and Arawi address the ethical burdens that arise from the need to respond to non-COVID-19 patients who are often left untreated during the pandemic.
2. *The moral obligation of healthcare workers amidst moral dilemmas:* Under this theme, Maraqa et al. adopt a “Mixed Method Study to Explore Ethical Dilemmas and Health Care Workers' Willingness to Work Amidst COVID 19 Pandemic in Palestine.” As the important role nurses play in the care of patients is often downsided, Alloubani et al. address the issue in their article the ethical role the nurses' play during the pandemic, finding among other conclusions that nurses in the survey believe nurses have an obligation to care for patients regardless of their medical diagnosis. Alahmad et al. tackle the “Ethical Challenges Related to the Novel Coronavirus (COVID-19) Outbreak” after conducting in-depth interviews with health professionals from Saudi Arabia.
3. *The fair allocation of scarce medical resources* appeared as another theme, with Huseynov et al. discussing the “General Public Preferences for Allocating Scarce Medical Resources during COVID-19.” In their “Fair allocation of the scarce medical resources, a comparative study from Jordan, Al Hussaini et al. surveyed physicians, medical students, allied health practitioners, religion scholars and laypeople revealing how each group prioritizes the distribution of resources. Using Fuzzy Logic, Saadah et al. propose a framework on “Whom Should Be Saved?” and Alhalaseh et al. discuss the allocation of already scarce medical resources arguing that solutions that might work in countries with limited resources are different from those usually adopted by the better-resourced countries.
4. *Clinical Trials*: This section highlights some of the major obstacles behind the continuity of clinical trials with Hashem et al. address the major obstacles behind the continuity of clinical trials in their article “Obstacles and Considerations Related to Clinical Trial Research during the COVID-19 Pandemic.” Abdelhafiz et al. raise the issue of factors affecting participation in trials in their article “Factors influencing participation in COVID-19 clinical trials: a multi-national study” and argue that during the pandemic, willingness to participate in clinical trials was affected by such factors as the scientific and ethical character of the trial, an opportunity to protect the family from the virus, access to additional healthcare, and the ability to return to community normalcy. Li et al. address the “Deficiencies in planning interventional trial registration of COVID-19 in China,” observing that the lack of appropriate planning resulted in over-registration of COVID-19 trials in China, with the result that the number of patients needed by the trials was actually greater than the number of newly-diagnosed patients.
5. *Psychological well-being*. The effect of the pandemic, lockdown, and quarantine on the psychological and mental well-being of various sectors in the community, and the ethical obligations toward those who are at risk formed the fifth theme. Guo et al. report the plethora of “Adverse psychological reactions and psychological aids for medical staff during the COVID-19 outbreak in China.” This is especially important as the experience of China was leading the world, although anxiety and the psychological impact were similar regardless of the geographic location. Saadeh et al. address this issue in her article on 6,157 Jordanian undergraduate university students, and how large increases in smartphone use becomes a concern, as 27.6 and 57.2% reported an increase and great increase, respectively, of their smartphone, with around 42% using theirs for more than 6 hours a day. Of interest, students' living environment proved significant in this study, as those, e.g., who lived in rural areas or in a home with a garden rather than in an apartment experienced lesser increases in their mobile phone use. Of interest, Yadav et al. also address the “Anxiety and Depression among Health Sciences Students at Home Quarantine during COVID-19 Pandemic in selected Provinces of Nepal” again addressing similarities of concerning issues in different countries. They too found, among other things, that factors such as place of residence significantly affected respondents' levels of depression and anxiety. Saaddeh et al. also look into the “Effect of COVID-19 quarantine on the sleep quality and the depressive symptom levels of university students in Jordan during the spring of 2020,” when the long-term lockdown was imposed on the country. The sleep quality of three-quarters of the participants was negatively affected by the extended quarantine. In addition, depressive symptoms were reported in 71% of participants, including 34% with moderate and 37% with high depressive symptoms scores. Meanwhile, Li et al. investigate the “Psychological distress, social support, coping style, and perceived stress among medical staff and medical students in the early stages of the COVID-19 epidemic in China.” Guo et al. elegantly performed a systematic review and meta-analysis on the “Depression and coping styles of college students during COVID-19 epidemic.” The number of articles under this theme clearly indicates the ethical obligation toward implementing effective measures to help mitigate the psychological effect of the imposed quarantine and lockdown, particularly among college and university students.
6. *The role states and governments played during the pandemic*. Some of these interventions were ethically disputed. Dave and Gupta address an essentially debatable issue of policies mandating tracking systems that were used during the pandemic and how ethical these were. Through deploying the Faden-Shebaya framework, which is used to justify public health interventions, the authors argue that while theoretically justified, it is difficult to defend a mandatory policy in practice. Freitas et al. write A reflection on the main ethical obstacles related to the strategic action “o brasil conta comigo.” Edlinger et al. ask “Is it legitimate for society to intervene in the way citizens live their lives when the cost of health care has to be borne by the general public?—General considerations and special implications during the Covid-19 pandemic.”
  Lastly, Odeh et al. came up with an interesting classification “iOntoBioethics: A Framework for the Agile Development of Bioethics Ontologies in Pandemics, Applied to COVID-19,” a unique and unprecedented work that will set the stage toward an artificial intelligence-based classification of the (bio)ethical published literature, which might contribute toward setting the stage for “Bioethics Informatics.”

It was an immense pleasure for us to co-edit this Frontier's issue. Nonetheless, bioethical dilemmas continue to arise almost every day along with ethical discussions, and debates. As long as the pandemic is still active, there will always be emerging new bioethical issues, the latest of which is related to the vaccine itself. We contend that several of the aforementioned themes can be extrapolated to fit discussions related to the vaccine, including the fair prioritization of this invaluable medical resource, the anxiety and hesitancy among some to receive the vaccine, and the role of governments in enforcing vaccination among its citizens.

We hope you will find this issue useful and enlightening.

## Author Contributions

MA-H, AM, TA, MZ, and HM contributed to the article and approved the submitted version.

## Conflict of Interest

The authors declare that the research was conducted in the absence of any commercial or financial relationships that could be construed as a potential conflict of interest.

## Publisher's Note

All claims expressed in this article are solely those of the authors and do not necessarily represent those of their affiliated organizations, or those of the publisher, the editors and the reviewers. Any product that may be evaluated in this article, or claim that may be made by its manufacturer, is not guaranteed or endorsed by the publisher.

